# Predicting wellbeing in empty-nesters: an ensemble machine learning approach with data-driven feature selection

**DOI:** 10.3389/fpsyg.2026.1888784

**Published:** 2026-07-29

**Authors:** Wael Khreich, Mona Ayoub

**Affiliations:** 1 Suliman S. Olayan School of Business, American University of Beirut, Beirut, Lebanon; 2 Department of Psychology, American University of Beirut, Beirut, Lebanon

**Keywords:** empty nest, wellbeing in later life, ensemble machine learning, feature selection, nested cross-validation, life satisfaction, happiness, meaning-making

## Abstract

Machine learning (ML) methods promise to enrich psychological research, yet worked examples that translate the logic of predictive modeling into psychological questions remain scarce. This study demonstrates a predictive ML workflow with ensemble feature selection, using the empty nest as its substantive case. Three research questions were addressed: whether empty-nest parents differ from parents whose children still live at home in happiness and life satisfaction (RQ1); which of 77 candidate predictors carry robust predictive signal for each wellbeing outcome (RQ2); and whether the same predictors operate in both groups (RQ3). European Values Study data (*N* = 43, 670 parents aged ≥45) were analyzed through propensity score matching, consensus feature selection across five complementary methods with nested cross-validation, and a three-phase group comparison. The groups showed virtually no difference in either wellbeing outcome, and this null result persisted after demographic matching. Because the design is cross-sectional, it concerns differences associated with empty-nest status rather than adjustment during the transition itself. Health status and perceived freedom/control emerged as the strongest predictors of happiness, whereas life satisfaction drew on a broader set spanning personal resources, social connections, institutional confidence, and social capital, a pattern partly consistent with Self-Determination Theory, particularly its autonomy and relatedness needs. Predictors overlapped substantially between groups, with only a modest subset of robust group-specific interaction effects. The findings suggest that wellbeing among parents in midlife and later adulthood depends on factors operating across family configurations rather than on empty-nest status, and they illustrate how predictive modelling can complement hypothesis-driven psychological research.

## 1 Introduction

Predictive machine learning (ML) methods can help address a common analytic challenge in psychological research: identifying which of many interrelated predictors carry robust signal beyond a particular sample or modeling choice. Yet worked examples that apply such methods to substantive psychological questions remain scarce. The present study offers one such example, using the empty-nest context and a large cross-national survey of parents in midlife and later adulthood. The period from midlife onwards (ages 45+) represents a pivotal period for wellbeing, marked by significant life transitions including career changes, evolving family roles, and preparations for later life (Mitchell and Lovegreen, 2009). Following Diener et al. (1999), wellbeing is conceptualized here as subjective wellbeing comprising two related but distinct components: *happiness*, the affective component reflecting a global evaluation of one's emotional experience, and *life satisfaction*, the cognitive component reflecting a deliberate judgement of one's life as a whole. Because the two components can diverge in their antecedents, they are examined as separate outcomes throughout this study.

The “empty nest,” when adult children leave the parental home, represents one such transition that has received considerable research attention due to its potential impact on parental wellbeing. Yet findings remain contradictory: while popular discourse often frames the empty nest as a period of loss and psychological distress (the so-called “empty nest syndrome”), empirical evidence presents a more nuanced picture spanning negative, null, and positive effects. One conceptual qualification frames the present investigation. The empty nest syndrome refers to distress during the *transition* period surrounding children's departure, not to permanently lowered wellbeing thereafter (Raup and Myers, 1989; Bouchard, 2014). Cross-sectional comparisons such as the present study therefore address a related but distinct question: whether wellbeing differences are associated with empty-nest *status* rather than with the transition itself. A null status difference cannot rule out transient adjustment difficulties; if such difficulties resolve ith time, however, any group differences should be concentrated among parents closer to the child-launching period, a pattern the age-stratified analyses reported below examine indirectly.

Empirical evidence on this question is strikingly contradictory, and the picture becomes clearer when the design and context of each study are considered. In an integrative review of predominantly Western studies, Bouchard (2014) concluded that the evidence does not support a universal empty nest syndrome, with children's departure yielding both positive and negative parental outcomes. Longitudinal designs, which can observe the transition itself, tend to find benign or positive effects: following parents in the German Aging Survey through the transition, Kristensen et al. (2021) found no change in depressive symptoms or loneliness after confound adjustment. In a nine-year prospective study of Australian midlife women, Dennerstein et al. (2002) found *improved* mood and fewer daily hassles once the last child left home. An exception is the German panel analysis by Piper and Jackson (2017), in which becoming an empty nester was followed by reduced life satisfaction, concentrated among men and unpartnered parents. Cross-sectional comparisons of empty-nest status yield more mixed results that vary with cultural context and statistical adjustment. On the null-to-positive side, Zhang (2020) found that Chinese empty-nest elders were no less happy than their non-empty-nest counterparts, with spousal absence and rural residence mattering instead, and Guo et al. (2016) reported *better* mental health among urban Chinese empty-nest elderly, largely reflecting their higher socioeconomic status. Anike (2023) found no significant association with life satisfaction among Nigerian middle-aged adults, and in a large Chinese national sample, Xu et al. (2023) found that initial health differences between groups disappeared entirely after multivariable adjustment. On the negative side, a meta-analysis of 29 Chinese studies (He et al., 2020) found lower quality of life among empty-nest elderly, and cross-sectional analyses of large Indian (Nayak et al., 2024) and Chinese (Wang et al., 2022) samples reported associations with depressive symptoms and cognitive impairment. Murugan et al. (2022) documented high loneliness among Indian empty-nest adults, albeit without a non-empty-nest comparison group. Living arrangements appear central to this heterogeneity: Yang et al. (2021) found worse cognitive function among Chinese empty-nesters living alone but *better* function among empty-nest couples, highlighting the protective role of partnership.

In sum, the contradictions largely track cultural context (collectivist societies show more negative effects), living arrangements (those living alone fare worse than couples; Piper and Jackson, 2017; Yang et al., 2021), and study design, as bivariate associations often attenuate or disappear after multivariable adjustment (Xu et al., 2023; Zhang, 2020) and longitudinal designs tend to find weaker effects than cross-sectional comparisons. Critically, no study has employed rigorous machine learning methods with proper cross-validation to identify robust predictors, nor has prior research systematically compared empty-nesters to non-empty nesters using data-driven feature selection.

Self-Determination Theory (SDT; Deci and Ryan, 2000; Ryan and Deci, 2017) provides the guiding theoretical lens for this investigation, positing three basic psychological needs: autonomy (freedom to make meaningful choices), relatedness (meaningful connections with others), and competence (sense of mastery and effectiveness). Although these needs were first studied as conditions for intrinsic motivation, SDT's basic psychological needs theory holds that their satisfaction is the proximal condition for psychological growth and wellness more broadly: circumstances that satisfy the three needs are theorized to promote wellbeing directly, whereas need frustration predicts ill-being (Ryan and Deci, 2017, 2001). Meta-analytic evidence confirms the association between need satisfaction and wellbeing specifically in later life (Tang et al., 2020), and cross-cultural research supports need satisfaction as a predictor of wellbeing across diverse contexts (Church et al., 2013). In the post-launch phase of family life, the three needs acquire specific content. Autonomy concerns the regained discretion over time, roles, and daily routines as child-rearing obligations recede. Relatedness concerns the reconfiguration of the parent–child bond alongside partner, friendship, and community networks as the child-centered household dissolves. Competence concerns mastery in the domains that structure this life stage: work or the transition out of it, the maintenance of health, and the parental role itself as it shifts from daily care to support at a distance. Complementary perspectives include meaning-making theories, which emphasize the role of spirituality and purpose during life transitions (Steger et al., 2009), and social capital and institutional trust theories, which highlight how confidence in societal institutions contributes to subjective wellbeing evaluations (Helliwell, 2004). However, prior research has not systematically tested which specific indicators across these domains most strongly predict wellbeing in this age range; this precision is critical for developing targeted interventions.

Although the departure of children is normatively a mid-adulthood event, empty-nest status in a cross-sectional sample of parents aged 45 and older spans the years from midlife well into old age. The relevant scope of the present investigation is therefore not the launching event itself but wellbeing across this extended post-launch phase, which connects the empty-nest literature to the broader study of wellbeing in aging. Understanding what predicts wellbeing across this phase is crucial for healthy aging, and it motivates the three research questions of this study: (RQ1) In cross-sectional data, do empty-nest parents differ from comparable parents whose children still live at home in their levels of happiness and life satisfaction? (RQ2) Which of the many candidate predictors available in a large values survey carry robust, generalizable predictive signal for each wellbeing outcome, and to what extent do the selected predictors align with or diverge from existing theoretical frameworks? (RQ3) Do the same predictors emerge for both groups, or are there group-specific pathways to wellbeing?

Answering these questions requires methods that can evaluate many correlated candidate predictors without capitalizing on chance, and traditional approaches face three interrelated problems here. First, limited predictor sets preclude comprehensive understanding. A recent scoping review (Park and Mendoza, 2022) found that most studies examine 5–10 variables selected for theoretical interest or data availability, rely on convenience samples, and adopt a theoretical approaches, potentially missing important predictors. Second, multicollinearity and overfitting plague regression models with many correlated predictors. Standard multiple regression with 25+ predictors yields unstable, non-replicable coefficients, a problem termed the “Table 2 Fallacy” (Westreich and Greenland, 2013). Third, lack of rigorous validation means findings often fail to replicate in independent samples (Yarkoni and Westfall, 2017). ML methods address these problems by changing the target of inference: rather than asking “what correlates with wellbeing in this sample?”, they ask “what predicts wellbeing in new, unseen data?” (Shmueli, 2010; Yarkoni and Westfall, 2017; Pargent et al., 2023; Schroeders et al., 2025). The logic rests on two ideas. Regularized regression methods such as elastic net deliberately shrink coefficients toward zero, trading a small bias for a large gain in stability; predictors that survive shrinkage are those carrying independent signal rather than noise or redundancy (Zou and Hastie, 2005). Cross-validation then provides repeated held-out evaluation: models are fitted on one part of the sample and evaluated on another, so that reported performance reflects how well the modeling pipeline generalizes to new observations rather than how well it fits the data at hand. A comparison with structural equation modeling (SEM) clarifies the division of labor. SEM is well suited to testing prespecified measurement and structural relations among theoretically specified constructs and remains the tool of choice for confirmatory theory testing. The present ML workflow serves a different purpose: exploratory prioritization among many correlated candidate predictors, with predictive performance evaluated on held-out data. It therefore complements, rather than replaces, confirmatory theory-testing approaches. In terms of the philosophy of science, the present approach operates in the context of discovery rather than justification (Shmueli, 2010): the goal is not to adjudicate causal mechanisms or to confirm a theory of empty-nest wellbeing, but to prioritize robust candidate predictors for subsequent theory-driven, longitudinal, and intervention-based research. Nor is the approach theory-free: the EVS item pool embodies decades of theory-guided measurement decisions, and the selected predictors are interpreted through theoretical lenses *post hoc*. The approach does not eliminate assumptions or replace theory; it reduces one form of researcher discretion, selective attention in predictor choice, within a pre-existing, theory-shaped measurement space.

The analytic workflow maps onto the research questions as follows. RQ1 is addressed through a **comparison group** design with **propensity score matching**, which balances the substantial demographic differences between empty-nest and non-empty-nest parents (especially age) before any comparison is made. RQ2 is addressed through **data-driven feature selection** starting from all 77 eligible predictors, using **ensemble consensus** across five selection methods: two penalized linear models (elastic net, LASSO), two tree ensembles that capture non-linearities and interactions (random forest, gradient boosting), and one model-agnostic permutation approach (each method is described in Section 2.4.2). Requiring agreement across methods with different assumptions, an established practice in ensemble feature selection (Saeys et al., 2008), reduces sensitivity to the assumptions of any single algorithm, and **nested cross-validation**, with all preprocessing and selection repeated within each fold to prevent data leakage, provides unbiased estimates of the modeling pipeline's out-of-sample performance. RQ3 is addressed through a **three-phase hybrid comparison** on the matched sample, testing whether the predictors identified for empty-nesters also operate for non-empty nesters. By comparing groups using a predictive ML workflow, this study aims to distinguish broadly shared wellbeing predictors from those whose relevance is more specific to empty-nest status. More broadly, it demonstrates an analytic approach for psychological questions involving many candidate predictors.

## 2 Materials and methods

### 2.1 Transparency and open science

This study follows best practices for machine learning in psychological research (Pargent et al., 2023; Yarkoni and Westfall, 2017). All analyses, including nested cross-validation procedures and hyperparameter tuning, were implemented in Python 3.13 using scikit-learn 1.6, pandas 2.2, and statsmodels 0.14 (for propensity score matching and statistical comparisons). Complete analysis code will be made available in a public repository upon acceptance. The European Values Study data are publicly available at https://search.gesis.org/research_data/ZA7503?doi=10.4232/1.14021. Because the study uses publicly available, de-identified secondary data, it was exempt from institutional review board (IRB) approval. Due to the iterative nature of model development, hypotheses were not pre-registered, but rigorous validation procedures were implemented to prevent overfitting and ensure reproducibility. Accordingly, the study is positioned as exploratory-predictive research operating in the context of discovery: it identifies robust predictive relationships whose causal interpretation requires subsequent confirmatory tests (Shmueli, 2010).

### 2.2 Participants and sampling

Data come from the European Values Study (EVS) Trend File 1981–2017, a large-scale, cross-national survey program (European Values Study, 2020). The EVS employs probability sampling with country-specific designs (stratified multistage random sampling) to ensure national representativeness. Cross-sectional sampling weights provided by EVS were applied in all analyses to account for sampling design and non-response. The EVS was chosen for its (1) large, representative samples across multiple countries, (2) comprehensive measurement of wellbeing and psychosocial predictors, and (3) high data quality standards with professional fieldwork and rigorous quality control.

Participants were included if aged ≥45 years and had at least one child (variable X011 > 0). Participants were classified into two groups based on whether children currently resided in the parental home (variable X012):

**Empty-nest group** (*n* = 23,568; 54.0%): parents aged ≥45 with at least one child, where no children currently reside in the parental home (X012 = 0). Mean age = 65.3 years (*SD* = 9.5); 57.0% female; 61.4% married/partnered; 56.4% retired.

**Non-empty-nest group** (*n* = 20,102; 46.0%): parents aged ≥45 with at least one child, where at least one child still resides in the parental home (X012 > 0). Mean age = 56.3 years (*SD* = 9.4); 54.3% female; 75.8% married/partnered; 23.9% retired.

**Group differences:** the groups differed substantially on demographics, with empty-nesters being older (*d* = 0.95), more likely to be retired, widowed (21.5% vs. 11.7%), and having lower income (*d* = −0.42). These demographic differences are addressed through propensity score matching (Step A) and controlled for in all analyses.

The analytic sample comprised *N* = 43, 670 adults from 42 countries and regions, predominantly European; note that the propensity-score-matched subsample used for group comparisons is substantially smaller (see Section 3.1.1). The largest national samples were Germany (*n* = 2, 586), Denmark (*n* = 2, 343), Czech Republic (*n* = 1, 872), Italy (*n* = 1, 865), Great Britain (*n* = 1, 573), and Spain (*n* = 1, 536). Non-European countries included the United States, Canada, Russia, Georgia, Armenia, and Azerbaijan. This comparison group design addresses a key limitation of prior empty-nest research, enabling distinction between predictors specific to the empty-nest experience and those reflecting general wellbeing patterns in this age range.

### 2.3 Measures

#### 2.3.1 Outcome variables

**Happiness** was assessed with a single item: “Taking all things together, would you say you are...” rated on a 4-point scale (1 = not at all happy to 4 = very happy; EVS variable A008_R, reverse-coded so higher = happier). Single-item happiness measures show adequate test-retest reliability (*r* = 0.64 over 2 years; Lucas and Donnellan, 2012) and correlate strongly with multi-item scales (*r* = 0.85; Cheung and Lucas, 2014). Empty-nest group: *M* = 3.02 (*SD* = 0.69); non-empty-nest group: *M* = 3.02 (*SD* = 0.69); Cohen's *d* = 0.00, indicating no group difference.

**Life satisfaction** was assessed with a single item: “All things considered, how satisfied are you with your life as a whole these days?” rated on a 10-point scale (1 = completely dissatisfied to 10 = completely satisfied; EVS variable A170). This item corresponds to the widely-used Satisfaction with Life Scale (Diener et al., 1985) and shows similar psychometric properties (Cheung and Lucas, 2014). Empty-nest group: *M* = 7.29 (*SD* = 2.19); non-empty-nest group: *M* = 7.14 (*SD* = 2.17); Cohen's *d* = 0.07, indicating a negligible group difference.

The correlation between happiness and life satisfaction was *r* ≈ 0.60, suggesting related but distinct constructs (affective vs. cognitive wellbeing; Diener et al., 1999). Notably, despite large demographic differences between groups, wellbeing levels were virtually identical.

#### 2.3.2 Predictor variables

All 77 eligible EVS predictor items, defined as those with < 20% missing data, were included without additional researcher-imposed theoretical pre-filtering; items with more missing data were excluded to avoid unstable imputation and to preserve comparability across group analyses. *Post hoc*, the predictors were organized into eight substantive domains; Table 1 lists every variable with its EVS code, and the codes are used in the Results to link text, tables, and figures unambiguously. The domains serve interpretive organization only; they are descriptive groupings, not validated multi-item scales or latent constructs. The domains comprise personal control and resources (perceived freedom and control over one's life, self-rated health, household income, and employment status); social connection (importance of family and of friends, generalized trust in people, marital status, memberships in social organizations, and national pride); and civic engagement (importance of leisure and of politics, political interest, and participation in political actions). Further domains cover spirituality and meaning (importance of religion, religious denomination and identity, service attendance, and religious beliefs); confidence in institutions (twelve items covering, among others, the education system, the police, parliament, and the social security system); and moral permissiveness (ten items on the justifiability of behaviors such as homosexuality, avoiding a public transport fare, cheating on taxes, or euthanasia). The remaining domains are social and economic values (tolerance toward various groups of neighbors, work importance and work-related attitudes, marriage attitudes, and economic and political attitudes) and demographics (age, sex, number of children, children at home, and age at completion of education).

**Table 1:** Predictor variables by domain.

Domain	Example variables	*n*
Control/resources	Freedom/control (A173), health status (A009_R), income (X047_EVS), employment status (X028)	4
Social connection	Trust in people (A165), national pride (G006_R), marital status (X007), social organization memberships (A065–A080), importance of family (A001_R), importance of friends (A002_R)	12
civic engagement	Employment status (X028), leisure importance (A003_R), politics importance (A004_R), political interest (E023_R), political actions (E025_R–E028_R)	8
Spirituality/meaning	Religion importance (A006_R), religious denomination (F024, F025), service attendance (F028_R), religious identity (F034_R), believe in hell (F053), God importance (F063)	7
Confidence in institutions	Churches, armed forces, education, press, unions, police, parliament, civil services, social security, companies, justice, EU (E069_01_R–E069_18_R)	12
Moral permissiveness	Justifiability of homosexuality, fare-dodging, tax cheating, bribery, euthanasia, suicide, soft drugs, etc. (F115–F126)	10
Social & economic values	Tolerance (A124_02, A124_06, A124_09), work importance (A005_R), work-related attitudes (C001–C019), marriage attitudes (D022), economic attitudes (E035–E039)	18
Demographics	Age (X003), age recoded (X003R, X003R2), sex (X001), number of children (X011), children at home (X012), education age (X023)	7
Total predictors		77

Domains overlap thematically; employment status (X028) is listed under both control/resources and civic engagement. Total unique predictors = 77.

All EVS items underwent rigorous cognitive pretesting and pilot studies to ensure cross-cultural equivalence (European Values Study, 2020). Variables with the _R suffix were reverse-coded such that higher values indicate more positive states (e.g., better health, more trust, and greater importance). Missing data among retained variables ranged from 0.0% (age, sex) to 19.8%. Missing values were handled via *k*-nearest neighbors imputation (*k* = 5) implemented separately for training and test sets in each cross-validation iteration to prevent data leakage (see Preprocessing below).

### 2.4 Analytic approach

#### 2.4.1 Overview

The analytic approach comprized three steps, described in detail below.

**Step A: propensity score matching**. The two groups differ substantially on demographics, especially age (*d* = 0.95). Without adjustment, any group difference in predictor effects could be an artifact of age or correlated confounds (retirement, income, marital status). Logistic regression estimated the probability of empty-nest status from age (X003), sex (X001), income (X047_EVS), education (X023, age at completion of education), and country (S009, one-hot encoded). Nearest-neighbor 1:1 matching without replacement was applied with a caliper of 0.2 standard deviations of the logit of the propensity score. Balance was verified by requiring |*SMD*| < 0.1 on all matching covariates. The matched sample was used for all group comparisons in Step C. To examine whether the null overall group effect masked heterogeneity, stratified comparisons (Cohen's *d* with 95% CI) were computed within age bracket, marital status, and employment subgroups on the matched sample.

**Step B: primary analysis (empty-nesters only)**. Because empty-nesters are the focal population, the full ensemble feature selection pipeline with nested cross-validation (100 outer iterations using ShuffleSplit 80/20, 10-fold inner CV) was run on the entire empty-nest sample (*n* = 23, 568) for each outcome. The full sample was used to maximize statistical power and generalizability; propensity score matching (Step A) discards approximately 60% of participants to achieve demographic balance, which is necessary for group comparisons but not for within-group prediction. This step identifies which predictors robustly predict wellbeing among empty-nesters and provides unbiased out-of-sample performance estimates. Step C then examines whether the same predictors emerge independently for both groups or whether group-specific pathways exist.

**Step C: hybrid comparison (three phases on matched sample)**. To test whether predictors differ between groups, a three-phase approach was applied to the matched sample from Step A:

*Phase 1: Separate Group Models*. Ensemble feature selection with 100 iterations of 5-fold cross-validation was run *separately* for each group. Features achieving consensus (≥60% of methods) in each group were categorized as shared (consensus in both groups), empty-nest-specific (consensus only in empty-nesters), or non-empty-nest-specific (consensus only in non-empty nesters).

*Phase 2: Descriptive Ranking of Group Differences*. Because Phase 1's binary consensus threshold can create spurious “group-specific” labels (a feature at 49% in one group and 51% in the other would appear group-specific despite near-identical importance), the group difference in selection frequency was quantified for each feature as a descriptive screening step. For each feature, mean selection frequencies were computed per iteration (averaging across the five folds), yielding 100 values per group, and features were ranked by the difference in mean selection frequency (in percentage points). Because the 100 ShuffleSplit iterations resample the same individuals and are therefore not fully independent, the assumptions of the independent-samples *t*-test are not strictly satisfied. The Benjamini-Hochberg-adjusted *t*-test results are therefore reported only as heuristic screening indicators and are not interpreted as confirmatory evidence; features passing this screen (adjusted *p* < 0.05) were flagged as candidates for the Phase 3 interaction tests, where inferential grounding for group differences is provided by the regularized interaction models.

*Phase 3: Targeted Interaction Tests*. For candidate features identified in Phase 2, predictor × group interactions were tested using elastic net on the combined matched sample. This targeted approach reduces multiple comparisons (from 77 to only candidates) while ensuring detection of true group-specific effects.

Following best practices for ML in psychology (Pargent et al., 2023; Schroeders et al., 2025), all preprocessing (imputation, scaling, encoding) and feature selection was conducted separately for training and test datasets to prevent information leakage. Consensus thresholding across five complementary selection methods reduced sensitivity to any single algorithm's assumptions by requiring convergent signal across linear, non-linear, and model-agnostic approaches.

#### 2.4.2 Ensemble feature selection

To reduce sensitivity to the assumptions of any single algorithm, ensemble consensus across five complementary methods was employed, following established practice in ensemble feature selection (Saeys et al., 2008): elastic net (L1/L2 regularization), LASSO (L1 regularization), random forest importance, gradient boosting importance, and permutation importance (model-agnostic, evaluated on held-out validation data). These methods span linear and non-linear approaches, ensuring that selected features are not artifacts of a single algorithm's assumptions. Features selected by ≥60% of methods (i.e., ≥3 out of 5) were retained for final modeling. Selection frequency (proportion of CV iterations where a feature achieved consensus) quantified stability across train-test splits. Two sensitivity analyses probed the robustness of this criterion: varying the consensus threshold from 40% to 80%, and recomputing consensus after leaving out each of the five methods in turn (Section 3.4).

#### 2.4.3 Elastic net regression for final models

After ensemble feature selection, elastic net models (Zou and Hastie, 2005) were trained on consensus-selected features. Elastic net combines L1 and L2 regularization penalties, handling multicollinearity via coefficient shrinkage while performing variable selection by driving some coefficients to exactly zero. It was chosen for final modeling due to its interpretability: standardized coefficients directly indicate effect direction and magnitude, unlike tree-based importance measures. Elastic net thus plays two distinct roles in the workflow: as one of the five selection methods within the ensemble, and as the final interpretable predictive model fitted to the consensus feature set. Hyperparameters (regularization strength λ, 8 values; L1/L2 mixing α, 11 values; 88 combinations) were tuned via inner cross-validation, selecting the combination that minimized root mean squared error (RMSE).

#### 2.4.4 Nested cross-validation

**Outer loop (100 iterations):** data were split 80% training / 20% test using ShuffleSplit to create independent iterations. Because ShuffleSplit samples with replacement across iterations, individual observations may appear in multiple test sets; consequently, the 100 test-set performance estimates are not fully independent, and reported standard deviations may slightly understate true uncertainty. Nevertheless, aggregating across 100 iterations yields stable average estimates.

**Feature selection split:** within each outer fold, the training data were further split 80/20 into a feature-selection training set and a validation set. The five ensemble methods were fit on the feature-selection training set; permutation importance was evaluated on the validation set. This additional split prevents feature selection from biasing inner-loop performance estimates.

**Inner loop (10-fold CV):** Hyperparameters were tuned via 10-fold cross-validation within the feature-selection training set, with the best combination selected via grid search based on minimum RMSE. The final model was then trained on the feature-selection training set with the optimal hyperparameters and evaluated on the held-out outer test set. The feature-selection validation set was deliberately excluded from hyperparameter tuning and final model fitting, so that no observation that influenced feature selection contributed to the final model. This conservative choice reduces the effective training sample to approximately 64% of the data in each outer iteration and, if anything, yields slightly conservative performance estimates.

**Preprocessing within folds:** Following Pargent et al. (2023), all transformations were fit only on training data:

1. Imputation: *k*-nearest neighbors (*k* = 5) using training data neighbors only
2. Variance filtering: remove zero-variance predictors (constant across training set)
3. Standardization: subtract training mean, divide by training SD: *x*_*std*_ = (*x* − μ_train_)/σ_train_

These trained transformations were then applied to test data, ensuring no information leakage from test to training.

#### 2.4.5 Performance metrics

Model performance was assessed using *R*^2^ (proportion of variance explained), RMSE (root mean squared error), and MAE (mean absolute error) on held-out test sets. Both training and test performance are reported; overfitting was quantified as $R_{\text{train}}^{2}-R_{\text{test}}^{2}$, following transparency recommendations (Pargent et al., 2023).

#### 2.4.6 Variable importance

For elastic net, standardized regression coefficients (β) were averaged across 100 outer iterations, computing mean, SD, and 95% confidence intervals. Selection frequency is defined as the proportion of iterations where a feature achieves consensus (≥60% of ensemble methods agree it is important). Higher selection frequency indicates more robust predictors that consistently emerge across different train-test splits.

#### 2.4.7 Software

All analyses were conducted in Python 3.13 using: pandas 2.2 (data manipulation), scikit-learn 1.6 (elastic net, preprocessing, cross-validation), statsmodels 0.14 (propensity score matching, statistical tests), matplotlib 3.9 and seaborn 0.13 (visualization), PyYAML 6.0 (configuration management). Outer cross-validation folds were executed in parallel across 8 cores for computational efficiency.

## 3 Results

### 3.1 Step A: propensity score matching and group comparisons (RQ1)

#### 3.1.1 Propensity score matching

Propensity score matching produced well-balanced samples for both outcomes. Of 23,568 empty-nesters and 20,102 non-empty nesters, 1:1 matching retained 9,204 pairs (39.1% of empty-nesters, 45.8% of non-empty nesters) for happiness and 9,320 pairs (39.5% and 46.4%, respectively) for life satisfaction. The substantial sample loss reflects the large age gap (*d* = 0.95) between groups, which limited propensity score overlap. Table 2 presents covariate balance before and after matching. All matching covariates achieved excellent balance after matching (|*SMD*| < 0.1). The largest pre-matching imbalance was age (*SMD* = 0.98), which was reduced to |*SMD*| < 0.08 after matching. Income imbalance (*SMD* = −0.42) was reduced to |*SMD*| < 0.06. In the matched happiness sample, empty-nesters reported *M* = 3.02 (*SD* = 0.69) and non-empty nesters *M* = 2.98 (*SD* = 0.71); for life satisfaction, empty-nesters reported *M* = 7.27 (*SD* = 2.14) and non-empty nesters *M* = 7.10 (*SD* = 2.22). These matched samples were used for all group comparisons in the hybrid analysis (Step C).

**Table 2:** Covariate balance before and after propensity score matching.

Outcome	Covariate	SMD before	SMD after	Improvement
*Happiness* (*n* = 9, 204 matched pairs)	Age (X003)	0.977	−0.076	0.901
	Sex (X001)	0.068	−0.001	0.067
	Income (X047_EVS)	−0.417	0.056	0.361
	Education (X023)	−0.018	−0.000	0.018
*Life satisfaction* (*n* = 9, 320 matched pairs)	Age (X003)	0.979	−0.074	0.905
	Sex (X001)	0.069	0.004	0.065
	Income (X047_EVS)	−0.419	0.059	0.360
	Education (X023)	−0.017	−0.005	0.012

SMD, Standardized Mean Difference. All post-matching |*SMD*| < 0.1, indicating excellent balance. Country (S009) was included in matching but omitted from table (one-hot encoded, all balanced).

#### 3.1.2 Group comparisons on wellbeing

Table 3 presents sample characteristics by group. Despite large demographic differences in the unmatched sample (empty-nesters were substantially older, *M* = 65.3 vs. 56.3 years, *d* = 0.95; more likely to be retired, 56.4% vs. 23.9%; lower income, *d* = −0.42), the two groups showed virtually *no difference* in wellbeing outcomes:

**Table 3:** Sample characteristics by group (*N* = 43,670).

Variable	Empty-nest (*n* = 23,568)	Non-empty-nest (*n* = 20,102)	Effect size (Cohen's *d*)
Demographics			
Age (years), *M* (SD)	65.3 (9.5)	56.3 (9.4)	0.95
Female (%)	57.0	54.3	–
Married/partnered (%)	61.4	75.8	–
Widowed (%)	21.5	11.7	–
Retired (%)	56.4	23.9	–
Income (1–10 decile), M (SD)	4.2 (2.50)	5.3 (2.60)	−0.42
Outcomes			
Happiness (1–4 scale), M (SD)	3.02 (0.69)	3.02 (0.69)	0.00
Life satisfaction (1–10), M (SD)	7.29 (2.19)	7.14 (2.17)	0.07

*d* = 0.2 small, *d* = 0.5 medium, *d* = 0.8 large. Unmatched sample.

Happiness was virtually identical across groups (empty-nest *M* = 3.02, non-empty-nest *M* = 3.02, *d* = 0.00), and life satisfaction showed only a negligible difference (empty-nest *M* = 7.29, non-empty-nest *M* = 7.14, *d* = 0.07).

These negligible differences persisted in the matched samples, where groups were balanced on demographics. The only substantial matched-sample differences were, by design, on children at home (X012, *d* ≈ −2.7) and number of children (X011, *d* ≈ −0.4). This finding directly addresses RQ1: empty-nest status has no meaningful association with wellbeing levels, regardless of whether demographic confounds are controlled through matching.

#### 3.1.3 Subgroup heterogeneity in group differences

Stratified analyses on the matched sample are reported in Table 4. Judged against Cohen's conventions (Cohen, 1988), no stratum-specific contrast reached even a small effect (|*d*| ≥ 0.2); individually, none of these values constitutes a noteworthy group difference, and most (all marital-status and employment strata, |*d*| ≤ 0.14) are best described as negligible. The informative result is instead the systematic direction pattern across age brackets: the group difference reversed sign with age. It was slightly negative among the youngest empty-nesters (ages 45–54: happiness *d* = −0.07, 95% CI [−0.12, −0.02]; satisfaction *d* = −0.06, [−0.11, −0.01]) and positive among older brackets (ages 65–74: happiness *d* = 0.17, [0.11, 0.24]; satisfaction *d* = 0.22, [0.15, 0.28]). Because the youngest bracket is plausibly, on average, closer to the child-launching period, this monotone gradient is consistent with the characterization of empty-nest distress as transitional adjustment followed by adaptation (see Introduction), although the small magnitudes and the absence of transition-timing data preclude strong claims. These patterns should additionally be interpreted with the caveat that propensity score matching was conducted on the full sample, not within strata; several strata showed residual covariate imbalance (e.g., age |*SMD*| > 0.1 in the 45–54 and 75+ brackets), which may partially confound the within-stratum comparisons.

**Table 4:** Subgroup heterogeneity: empty-nest vs. non-empty-nest group differences within strata (matched sample).

Outcome	Stratum	*n*_EN	*n*_NEN	*d*	95% CI	*p*	Bal.
Happiness: by age bracket	45–54	2,823	3,146	−0.07	[−0.12, −0.02]	0.005	!
	55–64	4,178	3,348	+0.08	[+0.03, +0.12]	0.001	
	65–74	1,808	1,881	+0.17	[+0.11, +0.24]	<0.001	
	75+	395	829	+0.18	[+0.06, +0.30]	0.004	!
Happiness: by marital status	Married/partnered	6,586	6,547	+0.07	[+0.04, +0.11]	<0.001	!
	Not married	2,600	2,631	+0.02	[−0.04, +0.07]	0.570	!
Happiness: by employment	Employed	4,751	4,522	+0.01	[−0.03, +0.05]	0.637	!
	Retired	3,060	3,213	+0.11	[+0.06, +0.16]	<0.001	!
	Other	1,342	1,414	+0.04	[−0.03, +0.12]	0.290	
Life satisfaction: by age bracket	45–54	2,873	3,191	−0.06	[−0.11, −0.01]	0.028	!
	55–64	4,193	3,418	+0.10	[+0.06, +0.15]	<0.001	
	65–74	1,866	1,882	+0.22	[+0.15, +0.28]	<0.001	!
	75+	388	829	+0.12	[−0.00, +0.24]	0.057	!
Life satisfaction: by marital status	Married/partnered	6,648	6,647	+0.11	[+0.07, +0.14]	<0.001	!
	Not married	2,655	2,652	+0.02	[−0.04, +0.07]	0.538	!
Life satisfaction: by employment	Employed	4,847	4,595	+0.04	[+0.00, +0.08]	0.034	!
	Retired	3,065	3,234	+0.14	[+0.09, +0.19]	<0.001	!
	Other	1,349	1,430	+0.01	[−0.06, +0.09]	0.731	

*d*, Cohen's *d* (empty-nest − non-empty-nest). Positive *d*, empty-nesters higher. Benchmarks: *d* = 0.2 small, *d* = 0.5 medium, *d* = 0.8 large (Cohen, 1988); all values below small. Bal. = “!” if any matching covariate has |*SMD*| > 0.1 within stratum (matching was performed on the full sample, not within strata). EN, Empty-Nest; NEN, Non-Empty-Nest.

### 3.2 Step B: ensemble feature selection and model performance (RQ2)

The primary ensemble machine learning pipeline, run on the empty-nest sample with nested cross-validation, yielded the following out-of-sample performance:

**Happiness** (*n* = 23, 568): $R_{test}^{2}=0.244\pm 0.012$, *RMSE* = 0.604, *MAE* = 0.464. Training performance ($R_{\text{train}}^{2}=0.246$) was virtually identical to test performance (overfit = 0.002), indicating minimal overfitting.

**Life satisfaction** (*n* = 23, 568): $R_{\text{test}}^{2}=0.362\pm 0.012$, *RMSE* = 1.751, *MAE* = 1.327. Again, negligible overfitting was observed (overfit = 0.000).

**Figure 1:**
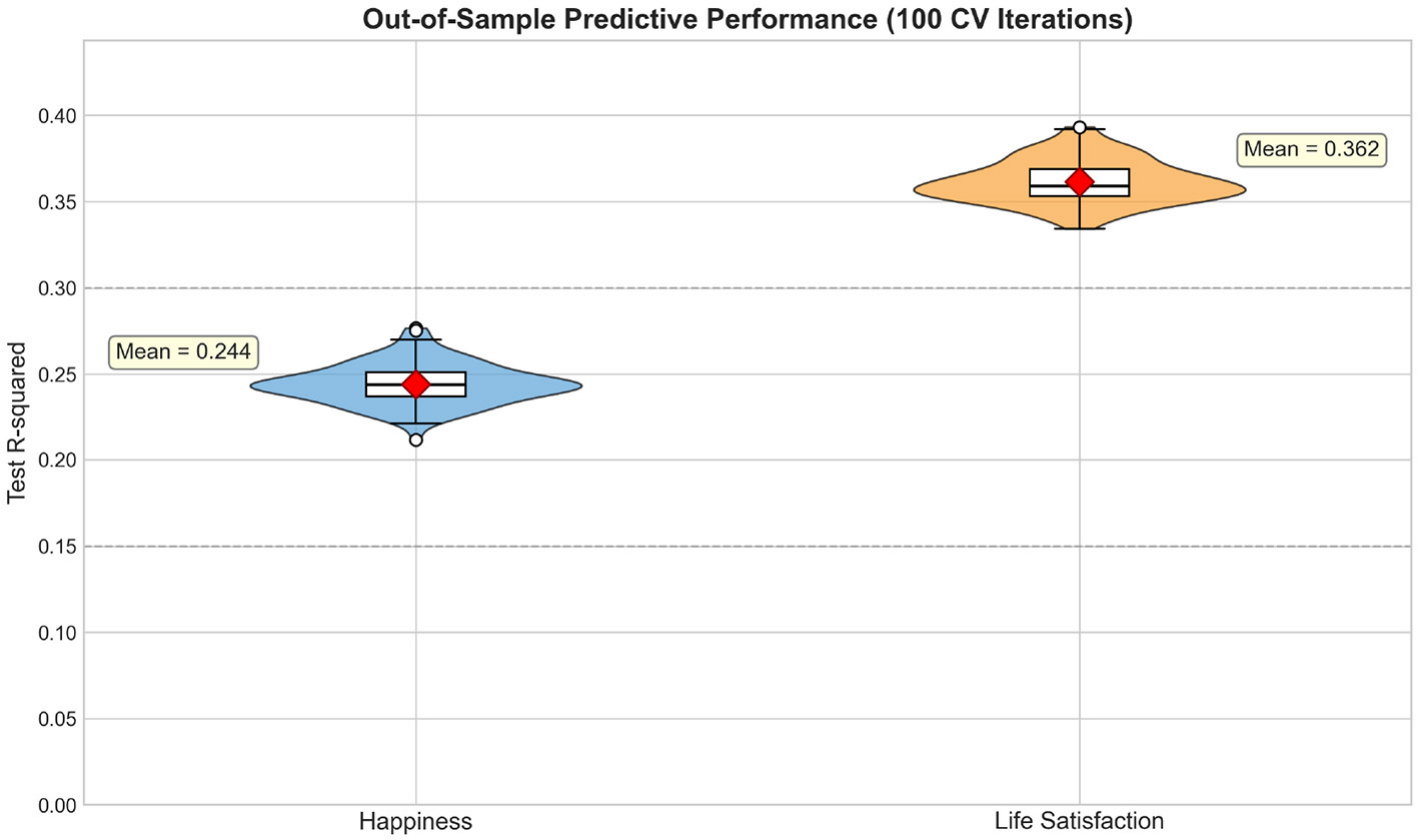
Model performance: distribution of *R*^2^ values across outer cross-validation iterations. Box plots show the distribution of test-set *R*^2^ for happiness **(left)** and life satisfaction **(right)** across 100 outer CV iterations. Life satisfaction shows higher explained variance (*R*^2^ = 0.36) than happiness (*R*^2^ = 0.24). Narrow distributions indicate stable performance. Empty-nest sample (*n* = 23, 568).

These *R*^2^ values are comparable to prior ML wellbeing studies: Oparina et al. (2024) report *R*^2^ ≈ 0.25–0.30 for life satisfaction, and Schroeders et al. (2025) report *R*^2^ ranging from 0.13 to 0.57 across psychological outcomes. The substantially higher explained variance for life satisfaction than happiness is consistent with the broader measurement range of the 10-point satisfaction scale. Table 5 presents the full performance summary; Figure 1 shows the distribution of *R*^2^ values across iterations.

**Table 5:** Model performance summary (empty-nest sample, nested cross-validation).

Metric	Happiness		Life satisfaction	
	Mean	SD	Mean	SD
*R*^2^ (train)	0.246	0.007	0.362	0.005
*R*^2^ (test)	0.244	0.012	0.362	0.012
RMSE (test)	0.604	0.007	1.751	0.022
MAE (test)	0.464	0.005	1.327	0.016
Overfit ($R_{train}^{2}-R_{test}^{2}$)	0.002	0.014	0.000	0.016

Production results (100 outer × 10 inner CV). *n* = 23, 568 empty-nesters. Overfit near zero indicates minimal overfitting.

**Figure 2:**
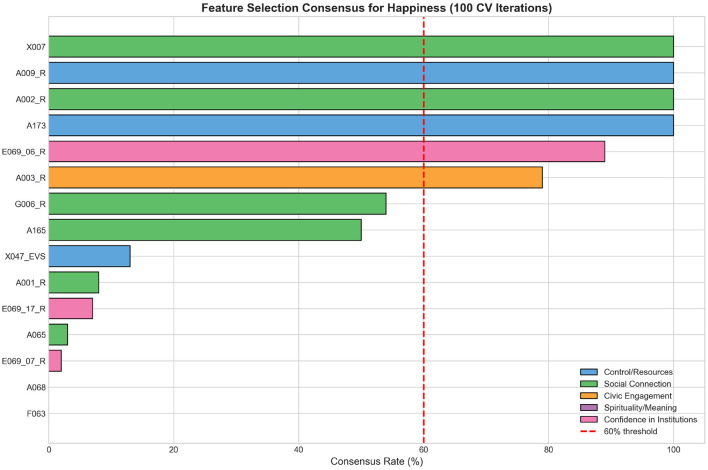
Variable importance for happiness. Ensemble feature selection frequencies for the top predictors of happiness among empty-nesters. Features achieving ≥60% consensus across five methods are shown. Health (A009_R) and freedom/control (A173) are the top predictors, alongside social connection variables (A002_R, X007). Empty-nest sample (*n* = 23, 568).

**Figure 3:**
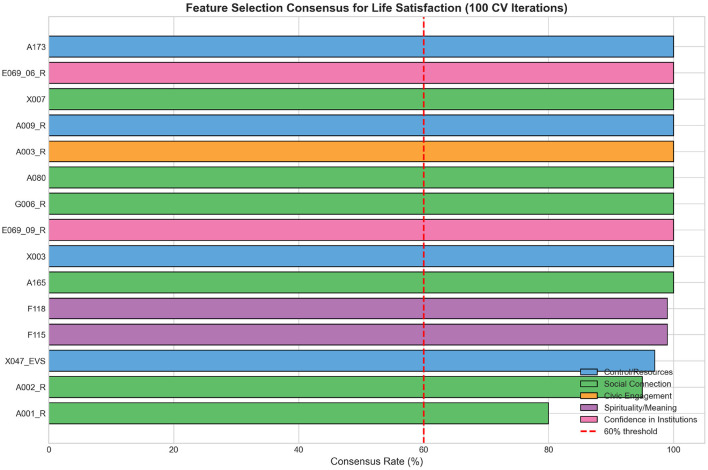
Variable importance for life satisfaction. Ensemble feature selection frequencies for the top predictors of life satisfaction among empty-nesters. Marital status (X007), freedom/control (A173), health (A009_R), and national pride (G006_R) achieve perfect selection frequency. Fifteen features achieved consensus, spanning personal resources, social connection, institutional confidence, and social capital. Empty-nest sample (*n* = 23, 568).

Starting with all 77 predictors, ensemble consensus (≥60% of five methods) identified markedly different predictor profiles for the two outcomes. Tables 6, 7 present the full feature selection results, and Figures 2, 3 visualize the selection frequencies. Appendix Tables A1, A2 provide extended rankings for the top 20 features.

**Table 6:** Ensemble feature selection results for happiness (empty-nest sample, *n* = 23, 568).

Rank	EVS code	Description	Sel. Freq.	Consensus
1	A009_R	Health status	1.00	Yes
2	A173	Freedom/control	0.99	Yes
3	A002_R	Friends importance	0.80	Yes
4	X007	Marital status	0.80	Yes
5	E069_06_R	Confidence in police	0.69	Yes
6	A003_R	Leisure importance	0.67	Yes
7	G006_R	National pride	0.55	No
8	A165	Trust in people	0.54	No
9	X047_EVS	Income level	0.43	No
10	E036	Private vs state ownership	0.40	No
11	X011	Number of children	0.40	No
12	X003	Age	0.40	No

Selection frequency = proportion of 100 outer CV iterations where feature achieved consensus (≥60% of 5 ensemble methods). Production results (100 × 10 CV).

**Table 7:** Ensemble feature selection results for life satisfaction (empty-nest sample, *n* = 23, 568).

Rank	EVS code	Description	Sel. Freq.	Consensus
1	X007	Marital status	1.00	Yes
2	A173	Freedom/control	1.00	Yes
3	A009_R	Health status	1.00	Yes
4	G006_R	National pride	1.00	Yes
5	E069_06_R	Confidence in police	1.00	Yes
6	A080	Belong to none	1.00	Yes
7	E069_09_R	Confidence in social security	0.99	Yes
8	A165	Trust in people	0.99	Yes
9	X003	Age	0.90	Yes
10	F118	Just.: Homosexuality	0.87	Yes
11	A003_R	Leisure importance	0.85	Yes
12	F115	Just.: Avoiding fare	0.83	Yes
13	A002_R	Friends importance	0.82	Yes
14	X047_EVS	Income level	0.78	Yes
15	A001_R	Family importance	0.63	Yes
16	F063	God importance	0.51	No
17	E069_13_R	Confidence in companies	0.44	No
18	E037	Govt. responsibility	0.44	No
19	E069_07_R	Confidence in parliament	0.43	No
20	E035	Income equality	0.42	No

Selection frequency = proportion of 100 outer CV iterations where feature achieved consensus (≥60% of 5 ensemble methods). Production results (100 × 10 CV).

**Happiness**. Six features achieved consensus: A009_R (health status, selection frequency 1.00), A173 (freedom/control, 0.99), A002_R (friends importance, 0.80), X007 (marital status, 0.80), E069_06_R (confidence in police, 0.69), and A003_R (leisure importance, 0.67). These predictors span multiple domains: personal resources (health, perceived control), social connection (friends, marital status), institutional confidence (police), and leisure. Notably, God importance (F063) appeared at rank 17 with a selection frequency of 0.40, below the consensus threshold, and religious organization membership (A065) was not selected by the ensemble.

**Figure 4:**
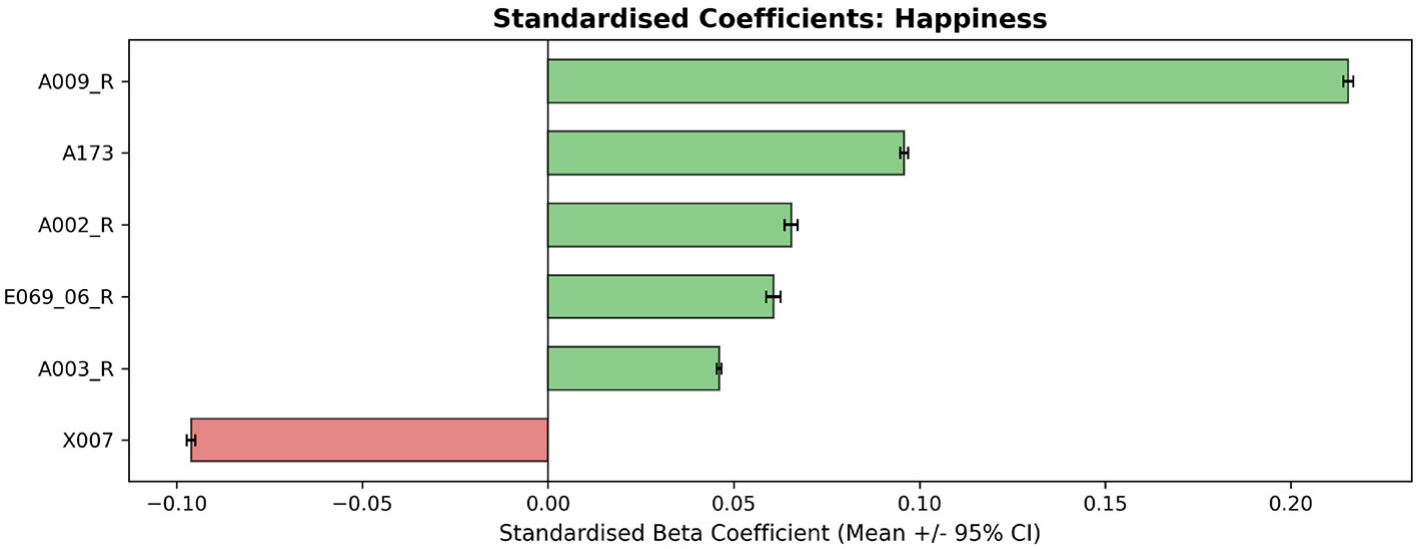
Standardized regression coefficients for happiness predictors. Mean standardized beta coefficients ($\bar{\beta }$) with 95% confidence intervals for all consensus features, averaged across 100 outer CV iterations. Positive values indicate higher predicted happiness. Empty-nest sample (*n* = 23, 568).

**Figure 5:**
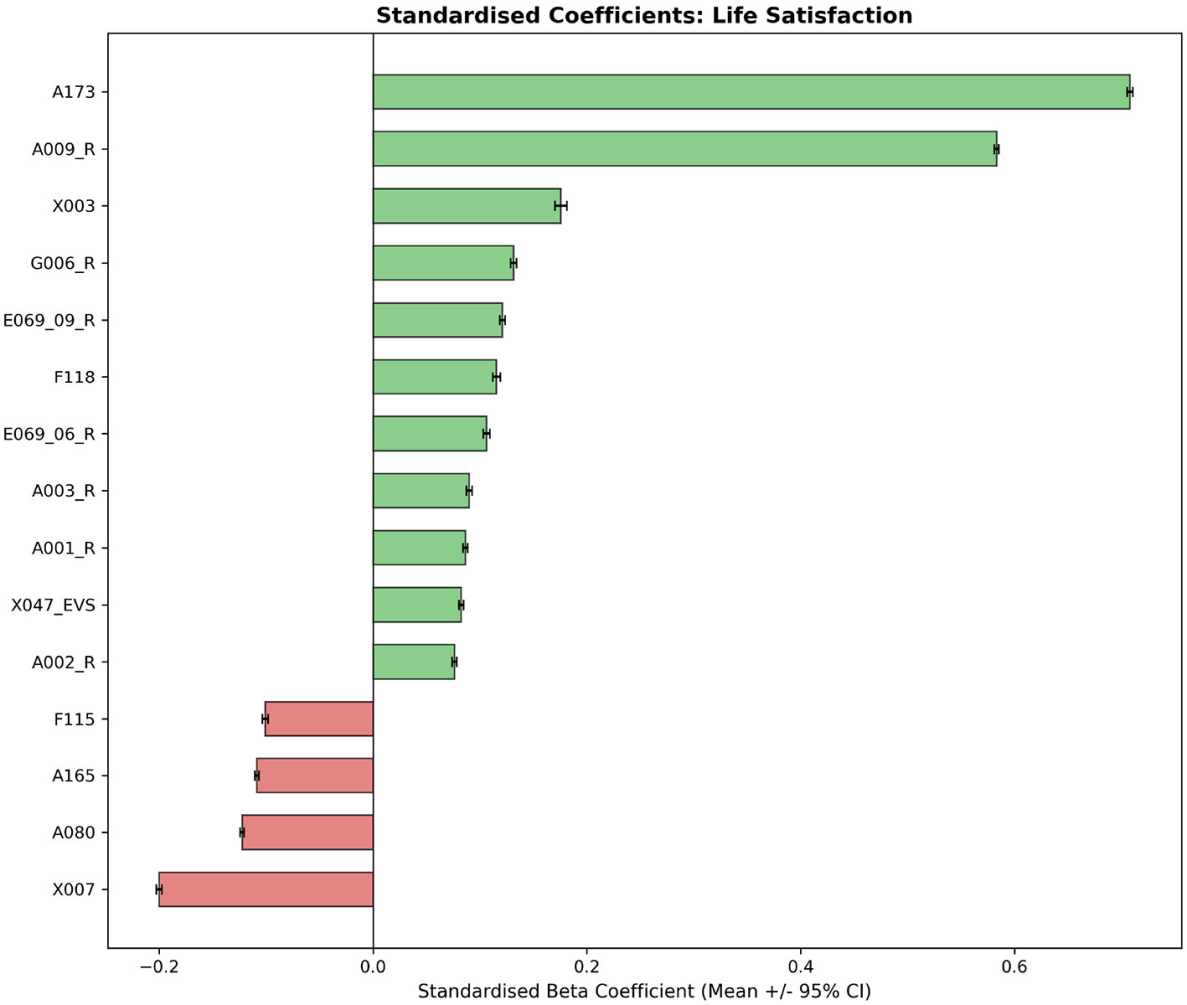
Standardized regression coefficients for life satisfaction predictors. Mean standardized beta coefficients ($\bar{\beta }$) with 95% confidence intervals for all consensus features, averaged across 100 outer CV iterations. Freedom/control (A173) and health (A009_R) show the largest coefficients. Empty-nest sample (*n* = 23, 568).

**Life satisfaction**. Fifteen features achieved consensus, reflecting the higher explained variance for this outcome. Four features achieved perfect or near-perfect selection frequency: X007 (marital status, 1.00), A173 (freedom/control, 1.00), A009_R (health, 1.00), and G006_R (national pride, 1.00). Institutional confidence variables followed closely: E069_06_R (confidence in police, 1.00) and E069_09_R (confidence in social security system, 0.99). Social connection variables also achieved consensus: A165 (trust in people, 0.99), A002_R (friends importance, 0.82), and A001_R (family importance, 0.63), along with A080 (belong to no organizations, 1.00). Demographic and resource variables included X003 (age, 0.90), X047_EVS (income, 0.78), and A003_R (leisure importance, 0.85). Two moral permissiveness variables achieved consensus: F118 (justifiable: homosexuality, 0.87) and F115 (justifiable: avoiding fare on public transport, 0.83), although God importance (F063) fell below the threshold at 0.51. Notably, all six happiness consensus features (A009_R, A173, A002_R, X007, E069_06_R, A003_R) also achieved consensus for satisfaction, indicating shared core predictors alongside outcome-specific additions. Table 8 and Figures 4, 5 present the standardized regression coefficients for all consensus features, quantifying each predictor's direction and magnitude. For happiness, health status had the largest coefficient ($\bar{\beta }=0.215$), followed by freedom/control and marital status ($|\bar{\beta }|\approx 0.096$ each). For life satisfaction, freedom/control ($\bar{\beta }=0.708$) and health ($\bar{\beta }=0.583$) dominated, with coefficients substantially larger than all other predictors. The theoretical implications of these predictor profiles are examined in the Discussion.

**Table 8:** Standardized regression coefficients for consensus features (empty-nest sample, *n* = 23, 568).

Feature	Description	$\bar{\beta }$	SD	95% CI
Happiness (6 consensus features)				
A009_R	Health status	0.215	0.007	[0.214, 0.217]
A173	Freedom/control	0.096	0.005	[0.095, 0.097]
X007	Marital status	−0.096	0.006	[−0.097, −0.095]
A002_R	Friends importance	0.065	0.009	[0.064, 0.067]
E069_06_R	Confidence in police	0.061	0.010	[0.059, 0.063]
A003_R	Leisure importance	0.046	0.003	[0.045, 0.047]
Life Satisfaction (15 consensus features)				
A173	Freedom/control	0.708	0.013	[0.705, 0.711]
A009_R	Health status	0.583	0.011	[0.581, 0.585]
X007	Marital status	−0.200	0.013	[−0.202, −0.197]
X003	Age	0.176	0.028	[0.170, 0.181]
G006_R	National pride	0.132	0.015	[0.129, 0.134]
A080	Belong to none	−0.122	0.010	[−0.124, −0.120]
E069_09_R	Confidence in social security	0.121	0.013	[0.119, 0.124]
F118	Just.: Homosexuality	0.116	0.018	[0.112, 0.119]
A165	Trust in people	−0.109	0.010	[−0.110, −0.107]
E069_06_R	Confidence in police	0.106	0.016	[0.103, 0.109]
F115	Just.: Avoiding fare	−0.101	0.013	[−0.103, −0.098]
A003_R	Leisure importance	0.090	0.013	[0.087, 0.092]
A001_R	Family importance	0.086	0.009	[0.084, 0.089]
X047_EVS	Income level	0.083	0.010	[0.081, 0.085]
A002_R	Friends importance	0.076	0.010	[0.074, 0.078]

$\bar{\beta }$ = mean standardized coefficient across 100 outer CV iterations. SD, Standard Deviation across iterations; CI, 95% Confidence Interval of the mean. Features sorted by $\left|\bar{\beta }\right|$ within each outcome. Negative coefficients reflect original EVS scoring direction (see Measures).

### 3.3 Step C: hybrid comparison (RQ3)

The three-phase hybrid comparison, conducted on the propensity-score-matched sample, addressed whether the same predictors emerge for both groups. Table 9 summarizes the results.

**Table 9:** Hybrid comparison: feature selection overlap between groups (matched sample).

Category	Happiness	Life satisfaction
Empty-nest consensus features	5	13
Non-empty-nest consensus features	6	16
Shared features	4	10
Empty-nest specific	1	3
Non-empty-nest specific	2	6
Shared features	A009_R (health status), A173 (freedom/control), X007 (marital status), A002_R (friends importance)	A009_R (health status), X003 (age), X007 (marital status), X047_EVS (income level), A173 (freedom/control), G006_R (national pride), E069_06_R (conf. police), E069_09_R (conf. social security), F063 (God importance), A003_R (leisure importance)
Empty-nest specific	X047_EVS (income level)	A165 (trust in people), A080 (belong to none), F118 (just.: homosexuality)
Non-empty-nest specific	E069_06_R (conf. police), A003_R (leisure importance)	A002_R (friends importance), E037 (govt. responsibility), E069_03_R (conf. education), A124_09 (tol.: homosexuals), F117 (just.: bribery), A124_06 (tol.: immigrants)
Phase 2: Candidate group differences (FDR-screened)	49 features	54 features
Phase 3: Robust interactions (>50% selection)	8 interactions	7 interactions

Based on propensity-score-matched samples. Phase 1 used 100 × 5 CV per group. Phase 2 ranked features by BH-FDR-screened selection-frequency differences.

**Phase 1: Separate group models**. Phase 1 evaluated whether the same variables emerged as important when the feature selection pipeline was run independently in each group. For happiness, the empty-nest group produced 5 consensus features and the non-empty-nest group produced 6, with 4 shared features (A009_R, A173, X007, A002_R). One feature was specific to empty-nesters (X047_EVS, income) and two were specific to non-empty nesters (E069_06_R, confidence in police; A003_R, leisure importance).

For life satisfaction, the empty-nest group produced 13 consensus features and the non-empty-nest group produced 16, with 10 shared features (A009_R, X003, X007, X047_EVS, A173, G006_R, E069_06_R, E069_09_R, F063, A003_R). Three features were specific to empty-nesters (A165, social trust; A080, belong to no organizations; F118, justifiable: homosexuality), and six were specific to non-empty nesters (A002_R, friends importance; E037, government responsibility; E069_03_R, confidence in education system; A124_09, tolerance of homosexuals; F117, justifiable: accepting a bribe; A124_06, tolerance of immigrants).

**Phase 2: Descriptive ranking of group differences**. Phase 2 evaluated whether the apparent group differences from Phase 1 were stable enough across resamples to be carried forward as candidates, rather than artifacts of the binary consensus threshold. Because repeated cross-validation splits are not fully independent, Phase 2 is interpreted as a stability-based screening procedure that identifies candidates for the Phase 3 interaction tests, rather than as confirmatory hypothesis testing. In this descriptive screening, most features showed group differences in selection frequency that passed the Benjamini-Hochberg FDR-adjusted threshold (*p*_*adj*_ < 0.05) used to rank and flag candidates: 49 of 77 features for happiness and 54 of 77 for life satisfaction. Because between-iteration variability in selection frequency is very small, standardized effect sizes for these differences take extreme values that cannot be interpreted against conventional benchmarks; group differences are therefore expressed as differences in mean selection frequency, in percentage points. Among significant features, the median absolute difference was 7.2 percentage points for happiness (interquartile range 2.4–13.7) and 6.3 for satisfaction (interquartile range 1.4–15.8). The largest differences were substantial: for happiness, income (X047_EVS) was selected in 79% of iterations among empty-nesters but only 44% among non-empty nesters, whereas tolerance of homosexual neighbors (A124_09) showed the reverse pattern (1% vs. 43%). For satisfaction, trust in people (A165; 91% vs. 28%) and belonging to no organizations (A080; 79% vs. 18%) were selected far more often among empty-nesters, whereas friends importance (A002_R; 45% vs. 100%) and tolerance of immigrant neighbors (A124_06; 11% vs. 83%) dominated among non-empty nesters. This pattern suggests that the *relative* importance of predictors differs between groups, even though the same core variables achieve consensus in both (Phase 1). All 49 (happiness) and 54 (satisfaction) candidate features were forwarded to Phase 3 for interaction testing.

**Phase 3: targeted interaction tests**. Phase 3 applied the most stringent criterion, testing which of the Phase 2 candidate differences survive when their predictor × group interaction terms compete alongside all predictor main effects within a single regularized model; only interactions that survive this competition indicate that a predictor's association with wellbeing differs by group after accounting for all other predictors. Elastic net models with predictor × group interaction terms (10 × 5 repeated K-fold CV) confirmed a subset of robust interactions (selected in >50% of folds). For happiness, eight interactions survived regularization: F116 (justifiable: cheating on taxes, 84%), A124_09 (tolerance of homosexuals, 78%), X047_EVS (income, 76%), F053 (believe in hell, 72%), E027_R (attending demonstrations, 72%), A124_06 (tolerance of immigrants, 60%), F126 (justifiable: taking soft drugs, 56%), and F123 (justifiable: suicide, 52%). For life satisfaction, seven robust interactions emerged: X007 (marital status, 100%), X001 (sex, 94%), A080 (belong to no organizations, 80%), E035 (income equality, 74%), F122 (justifiable: euthanasia, 72%), E026_R (joining boycotts, 60%), and E039 (competition good/harmful, 58%). Table 10 reports the full interaction coefficients.

**Table 10:** Robust interaction effects: predictor × empty-nest status (phase 3).

Outcome	Variable	Sel.Freq	Coef	Direction
Happiness	F116 (just.: cheating on taxes)	0.84	0.0035	Stronger
	A124_09 (tolerance of homosexuals)	0.78	0.0050	Stronger
	X047_EVS (income)	0.76	0.0040	Stronger
	F053 (believe in hell)	0.72	−0.0049	Weaker
	E027_R (attending demonstrations)	0.72	0.0079	Stronger
	A124_06 (tolerance of immigrants)	0.60	−0.0019	Weaker
	F126 (just.: taking soft drugs)	0.56	−0.0015	Weaker
	F123 (just.: suicide)	0.52	−0.0021	Weaker
Satisfaction	X007 (marital status)	1.00	−0.0424	Weaker
	X001 (sex)	0.94	0.0230	Stronger
	A080 (belong to none)	0.80	−0.0125	Weaker
	E035 (income equality)	0.74	0.0098	Stronger
	F122 (just.: euthanasia)	0.72	0.0108	Stronger
	E026_R (joining boycotts)	0.60	0.0069	Stronger
	E039 (competition good/harmful)	0.58	0.0096	Stronger

SelḞreq proportion of CV folds with non-zero interaction coefficient. Only robust interactions (>50% selection) shown. Direction: “Stronger”, predictor effect larger for empty-nesters. Based on elastic net with 10 × 5 repeated K-fold CV.

Taken together, the hybrid comparison reveals a nuanced picture: the core predictors of wellbeing are shared across family configurations (Phase 1), but the *relative importance* of many predictors appears to differ between groups at the descriptive screening stage (Phase 2). However, when tested via regularized interaction models (Phase 3), only a modest subset of these statistical differences translated into robust predictor × group interactions, suggesting that the practical effect of group membership on predictor relevance is concentrated in specific domains (moral permissiveness and tolerance for happiness; demographics and economic attitudes for life satisfaction).

### 3.4 Consensus threshold sensitivity

To assess the robustness of feature selection, a sensitivity analysis varied the consensus threshold from 40% to 80% of ensemble methods (compared with the primary 60% threshold). For happiness, the same six features achieved consensus at thresholds 0.4–0.6, dropping to four at thresholds 0.7–0.8 (E069_06_R and A003_R dropped), indicating that the core predictors (A009_R, A173, A002_R, X007) are robust across different consensus definitions. For life satisfaction, 15 features achieved consensus at thresholds 0.4–0.6, and 14 features at thresholds 0.7–0.8 (only A001_R dropped at the most stringent thresholds). This stability across thresholds provides evidence that the identified predictor sets are not artifacts of the particular consensus criterion chosen.

A complementary leave-one-method-out analysis recomputed the consensus sets after removing each of the five selection methods in turn, addressing whether any single algorithm drives the results. The consensus sets proved highly stable (Jaccard similarity with the full five-method ensemble 0.75–1.00 for happiness and 0.93–1.00 for satisfaction). The four core happiness predictors (A009_R, A173, A002_R, X007) were retained in all five reduced ensembles; removing a tree-based method dropped only the weakest consensus feature (A003_R, leisure importance), and removing LASSO, the most conservative method, slightly enlarged the set (adding A165, trust in people, and G006_R, national pride). For satisfaction, at least 14 of the 15 consensus features were retained in every reduced ensemble, with only the weakest feature (A001_R, family importance) sensitive to method removal. The consensus sets are thus not driven by any single algorithm, supporting the use of a methodologically diverse ensemble.

## 4 Discussion

Using a comparison group design with propensity score matching and ensemble machine learning in a large European sample, this study examined whether empty-nest status is associated with persistent wellbeing differences and whether wellbeing predictors differ between empty-nest and non-empty-nest parents. Three key findings emerged.

### 4.1 Empty-nest status has minimal association with wellbeing

The most striking finding is the *absence* of a meaningful difference in wellbeing between groups. Despite substantial demographic differences, empty-nesters and non-empty nesters reported essentially identical happiness and life satisfaction (Table 3), and these negligible differences persisted after propensity score matching balanced the groups on age, sex, income, education, and country (Table 2). These results do not support the notion, prevalent in both popular discourse and early research (Raup and Myers, 1989), that empty-nest status entails lasting wellbeing deficits; as noted in the Introduction, the transitional adjustment claim at the heart of the syndrome concept is not testable with cross-sectional status comparisons.

These findings align with recent prospective studies finding no consistent negative effects of the empty-nest transition (Kristensen et al., 2021; Zhang, 2020; Bouchard, 2014). The absence of group differences in wellbeing, combined with substantial overlap in core predictors between groups, suggests that empty-nest status is not associated with lower wellbeing in this cross-sectional comparison. Rather, wellbeing appears to depend primarily on factors that operate regardless of whether children have left home, though with some variation in relative importance across family configurations.

The stratified analyses add nuance without altering this conclusion (Table 4). No stratum-specific contrast reached even a small effect by Cohen's conventions, so none constitutes a noteworthy group difference in its own right.

What merits attention is the direction pattern across age: the group difference reversed sign from slightly negative among the youngest empty-nesters to slightly positive among older brackets. Because the youngest bracket is plausibly, on average, closer to the child-launching period, this gradient is consistent with the theoretical characterization of empty-nest distress as transitional adjustment followed by adaptation, and possibly eventual net gains (Mitchell and Lovegreen, 2009). On this reading, any distress is concentrated closer to the transition, a hypothesis that requires longitudinal testing. Tentative direction patterns within marital-status and employment strata (slightly more positive contrasts among partnered and retired empty-nesters) would be consistent with partnership buffering the transition (Piper and Jackson, 2017; Yang et al., 2021) and with a “double freedom” interpretation in which the simultaneous absence of work and childcare obligations facilitates self-directed activity. Given the negligible magnitudes, however, these readings remain speculative.

The regularized interaction analysis (RQ3) provides insight into which of these apparent moderations reflect genuine group-specific effects versus confounded associations. Age × group was not selected for either outcome, suggesting that the descriptive age-related pattern is accounted for by correlated predictors such as health status, income, and retirement. In contrast, marital status × group was consistently selected for satisfaction across cross-validation folds, indicating that partnership's moderating role persists after controlling for all other predictors. Employment status × group was not robustly selected for either outcome, suggesting that the descriptive pattern among retirees is largely explained by correlated variables. This divergence between descriptive and conditional findings illustrates a key advantage of the combined approach: descriptive subgroup analysis reveals the raw patterns that clinicians and policymakers may observe, while regularized ML identifies which patterns reflect independent moderation versus confounded associations.

### 4.2 Health and autonomy dominate both outcomes; broader predictors for satisfaction

The parsimonious six-feature happiness model centered on health and perceived control, complemented by social connections (friends importance, marital status) and institutional confidence (see Tables 6, 8 for full details). Life satisfaction drew on a substantially broader fifteen-feature set that additionally incorporated institutional trust, social capital, demographic resources, and moral permissiveness variables (Tables 7, 8). Notably, no religiosity variables achieved consensus for either outcome, contrary to some prior findings linking religiosity to wellbeing. This partial divergence between outcomes reinforces the theoretical distinction between affective and cognitive wellbeing (Diener et al., 1999) and suggests that interventions targeting life satisfaction may need to address a broader range of psychological, institutional, and contextual factors than those targeting happiness.

### 4.3 Alignment with theoretical frameworks

The selected predictors can be interpreted post hoc in relation to SDT and complementary frameworks, providing a theory-informed account of which domains carry robust predictive signal; this interpretation is necessarily indirect, because the EVS does not include a dedicated basic-need-satisfaction scale. The clearest alignment with SDT concerns autonomy: freedom/control achieved near-perfect consensus for both outcomes and was the largest standardized predictor of life satisfaction (Table 8), underscoring the centrality of perceived control for adults in this age range (Deci and Ryan, 2000; Ryan and Deci, 2017). Relatedness is also well represented among the selected predictors, with friends importance and marital status achieving consensus for both outcomes, and satisfaction additionally drawing on family importance and interpersonal trust. Competence is less directly assessed in the EVS. Health status, which emerged as the top predictor of happiness and achieved perfect consensus for satisfaction, is best viewed as a major wellbeing resource in its own right (DeNeve and Cooper, 1998) and only indirectly as a reflection of stage-salient capability; it should not be treated as a direct measure of competence satisfaction.

Religion and spirituality variables did not achieve consensus for either outcome, contrasting with meaning-making perspectives (Steger et al., 2009) and possibly reflecting the secular orientation of many European countries in the EVS sample. The moral permissiveness variables that did achieve consensus for satisfaction (justifiable: homosexuality, avoiding fare) measure tolerance rather than religiosity per se. Institutional confidence variables and national pride achieved consensus for satisfaction, aligning with social capital and institutional trust theories (Helliwell, 2004); this macro-level dimension is not captured by SDT's focus on interpersonal need satisfaction but contributes substantially to cognitive wellbeing evaluations.

Taken together, SDT offers a useful interpretive foundation for understanding wellbeing in this population, particularly through autonomy and relatedness, but a comprehensive account requires integrating health, institutional trust, and social capital; these alignments are interpretive rather than confirmatory tests of SDT.

### 4.4 Shared core with group-specific moderation

The three-phase hybrid comparison revealed that the core predictors of wellbeing are shared across family configurations, but with meaningful variation in relative emphasis. The shared core (health, autonomy, and social connection) suggests that wellbeing interventions targeting these domains should benefit adults regardless of family configuration. However, regularized interaction models identified a modest subset of robust group-specific effects concentrated in moral permissiveness and tolerance (happiness) and demographics and economic attitudes (satisfaction), indicating that some tailoring may be warranted. Importantly, the predictors identified in the primary analysis (Step B) are not artifacts of the empty-nest experience; the core variables operate in both groups.

### 4.5 Scope of predictive models

The models explained roughly a quarter of the variance in happiness and slightly more than a third in life satisfaction (Section 3.2), leaving most of the variance unaccounted for. While this performance is comparable to prior ML wellbeing studies (Oparina et al., 2024), the unexplained variance warrants discussion. Several factors likely contribute. First, the EVS lacks personality measures; the Big Five traits alone typically account for 10–15% of wellbeing variance in regression models (DeNeve and Cooper, 1998), and their absence likely inflates the unexplained proportion. Second, single-item outcome measures introduce substantial measurement error; attenuated reliability mechanically limits the maximum achievable *R*^2^. Third, momentary situational factors (mood at survey completion, recent life events) add noise that stable predictors cannot capture. Fourth, country-level variance in institutional quality, social norms, and economic conditions may influence wellbeing through channels not fully represented by individual-level predictors. These considerations suggest that the identified predictors are likely more important than the explained-variance figures alone imply, and that the upper bound of predictability with survey data and single-item outcomes lies far below its nominal maximum.

### 4.6 Practical implications

**For clinicians:** The absence of meaningful group differences suggests that empty-nest status alone should not be treated as a risk marker for poor wellbeing; health, perceived control, social connection, and recency of the transition are more informative considerations. Attention to parents in the years immediately surrounding children's departure nevertheless remains warranted, given the transitional character of the phenomenon. Practitioners should prioritize health status and perceived freedom/control as the strongest modifiable predictors (Table 8), alongside social connections (friends, marital quality). The standardized coefficients for perceived control and health were substantially larger than those of all other predictors, suggesting clear clinical relevance.

**For policy:** institutional confidence (police, social security) and national pride emerged as strong predictors of life satisfaction, suggesting that trust in public institutions contributes to citizens' cognitive wellbeing evaluations. Health promotion and policies supporting perceived autonomy remain important across family configurations.

**For researchers:** the comparison group design with propensity score matching demonstrates the value of including balanced control groups in empty-nest research. Without the non-empty-nest comparison, findings from the primary analysis could have been incorrectly attributed to the empty-nest experience rather than recognized as universal patterns among adults in this age range.

### 4.7 Machine learning as a complement to hypothesis-driven psychology

Beyond its substantive findings, this study illustrates a workflow that psychological researchers can adopt when the number of plausible predictors exceeds what conventional models handle well. The benefits are threefold. First, regularized models with ensemble consensus can screen large, correlated predictor sets without producing the unstable coefficients that afflict standard multiple regression (Westreich and Greenland, 2013; Zou and Hastie, 2005). Second, nested cross-validation provides repeated held-out evaluation of every analytic step, yielding estimates of how well the modeling pipeline generalizes to new observations rather than how well it fits the sample at hand (Yarkoni and Westfall, 2017; Pargent et al., 2023). Third, out-of-sample performance keeps theoretical claims accountable: a predictor that does not improve prediction in held-out data is unlikely to be practically important, however statistically significant it may appear in-sample (Shmueli, 2010). In the present study, this workflow served a specific purpose: it helped separate predictors broadly relevant to wellbeing among parents in midlife and later adulthood from candidate group-specific moderators. Without the comparison group and predictive screening, general wellbeing predictors such as health, perceived control, and social connection could easily have been overinterpreted as features of the empty-nest experience itself.

These benefits come with clear boundaries, and established alternatives remain preferable for other goals. Predictive performance in held-out data shows that a set of variables carries generalizable signal for wellbeing; it does not show that changing those variables would change wellbeing, nor does it establish the temporal ordering of empty-nest status, health, perceived control, and social relationships. Structural equation modeling is the method of choice when the aim is to test a prespecified theoretical structure, including measurement models and mediation pathways, among a limited number of constructs. Preregistered confirmatory regression is preferable when a small number of hypotheses derive directly from theory and the predictor set is correspondingly compact. Multiverse and specification-curve approaches address a different problem, the sensitivity of conclusions to analytic decisions, and can complement either mode. The natural division of labor is sequential: data-driven screening of the kind demonstrated here establishes which theoretically motivated predictors carry independent, generalizable signal; confirmatory designs then test the causal claims that those predictors suggest. Used this way, machine learning does not replace hypothesis-driven psychology but supplies it with better-vetted hypotheses.

### 4.8 Limitations

**Cross-sectional design:** causal direction cannot be established, and, because the empty nest syndrome is theorized as transitional distress, the present design cannot test the syndrome itself; it tests whether empty-nest status is associated with persistent wellbeing differences. Longitudinal research tracking parents before and after children leave would clarify whether the transition causes temporary or lasting wellbeing changes or whether pre-existing differences explain group composition.

**Residual confounding:** despite propensity score matching, residual confounding from unmeasured cohort effects may persist. The matched samples achieved excellent balance (|*SMD*| < 0.1) on observed covariates, but unobserved confounds cannot be ruled out.

**Empty-nest operationalization:** the binary classification (children at home vs. not) is necessarily coarse. The EVS does not capture the timing of the transition, the reasons for children's departure or continued co-residence, whether children have returned after an initial departure (“boomerang” children), or whether the empty nest was voluntary (successful child-launching) or involuntary (estrangement, bereavement). These distinctions may moderate the relationship between empty-nest status and wellbeing. Future research with richer data should examine these subgroups separately.

**Propensity score matching sample loss:** matching retained approximately 39% of empty-nesters and 46% of non-empty nesters, discarding roughly 60% of the sample. While the matched samples achieved excellent covariate balance, this substantial trimming limits external validity; the matched-sample results apply primarily to the subset of parents whose propensity scores overlapped across groups. Moreover, propensity score matching was conducted on the full sample rather than within subgroups; consequently, the stratified subgroup comparisons (Table 4) may exhibit residual covariate imbalance within certain strata (e.g., age |*SMD*| > 0.1 in the youngest and oldest brackets), and these within-stratum effect sizes should be interpreted with appropriate caution.

**Unmeasured personality:** the EVS lacks Big Five personality measures. Personality traits may explain apparent predictor effects; for example, neuroticism may lower both perceived control and wellbeing, inducing an association between them.

**Cultural generalizability:** although the sample spans 42 countries including several non-European nations, the majority of participants are from Western and Northern European contexts. Results may not generalize to East Asian or other collectivist cultures where multigenerational households are normative and the “empty nest” construct carries different connotations. Moreover, within the European sample, cultural variation across sub-regions (Mediterranean, Nordic, and post-Soviet) may moderate predictor effects in ways not captured by the present individual-level analysis.

**Single-item outcome measures:** both wellbeing outcomes were measured with single items, introducing measurement error. Multi-item measures would provide more reliable assessment.

**Indirect measurement of theoretical constructs:** the EVS does not include a dedicated basic-psychological-need-satisfaction scale, so the SDT interpretation is necessarily indirect. The clearest mapping is to autonomy (perceived freedom/control) and relatedness (friends and family importance, marital status, interpersonal trust); competence is represented only indirectly through health, work, and resource indicators and could not be assessed directly.

**Clustering:** observations within countries are not independent, and this clustering may affect cross-validation performance estimates. Country was included as a covariate in propensity score matching to address cross-national confounding, but the nested cross-validation procedure did not account for the clustered data structure, which may lead to slight underestimation of prediction error variability.

### 4.9 Future directions

The cross-sectional design represents the most pressing limitation to address: longitudinal studies tracking parents before and after children leave home would clarify whether the null group effect reflects genuine resilience or pre-existing selection into empty-nest status. Such studies would also show whether the subgroup heterogeneity observed here (e.g., younger empty-nesters faring worse) reflects transient adjustment difficulties or stable differences. Such designs should incorporate Big Five personality measures, whose absence from the EVS likely accounts for a substantial portion of unexplained variance and may confound apparent predictor effects (e.g., neuroticism influencing both perceived control and wellbeing). Replication in non-European, particularly collectivist, cultures is also warranted, given that multigenerational co-residence is normative in many East Asian contexts and the “empty nest” construct may carry different psychological meaning. Beyond replication, intervention studies could test whether strengthening the core predictors identified here, particularly perceived autonomy, health, and social connections, yields measurable wellbeing improvements for empty-nesters. Finally, the EVS does not capture when children left home; future research with richer data should examine how transition recency moderates outcomes, distinguishing recent empty-nesters (who may still be adjusting) from those who transitioned years earlier.

## 5 Conclusion

Using a comparison group design with propensity score matching and ensemble machine learning in a large European sample, this study found that empty-nest status has virtually no association with wellbeing. Despite large demographic differences between empty-nesters and non-empty nesters, the groups showed essentially identical happiness and life satisfaction, and these negligible differences persisted after propensity score matching.

The ensemble feature selection approach revealed that happiness and life satisfaction share core predictors but differ in breadth. The parsimonious happiness model centered on health and freedom/control alongside social connections. Satisfaction drew on a wider set of predictors including institutional confidence, social capital, and demographic resources, with freedom/control and health as the dominant predictors. A three-phase hybrid comparison found that core predictors are shared across family configurations, though regularized interaction models identified a modest number of group-specific effects concentrated in moral permissiveness and tolerance (happiness) and demographics and economic attitudes (satisfaction).

These findings do not support the notion that empty-nest status is associated with lasting wellbeing deficits; whether the transition itself is temporarily distressing remains a question for longitudinal designs. The same core factors (health, autonomy, social connection, and institutional trust) predict wellbeing across family configurations, albeit with some variation in relative emphasis. For practitioners, these findings suggest that empty-nest status alone is not a useful risk marker for poor wellbeing. Instead, supporting physical health and perceived control for emotional wellbeing, and fostering autonomy, social connections, and institutional trust for life satisfaction, may benefit all adults aged 45 and older, with modest additional attention to group-specific domains where interaction effects were observed.

Methodologically, this study demonstrates the value of propensity score matching combined with predictive ML methods in empty-nest research. Without the comparison group, findings from the primary analysis could have been incorrectly attributed to the empty-nest experience rather than recognized as universal patterns. Future research should employ longitudinal designs, include personality measures, and test whether these findings generalize to non-European cultures.

## Data Availability

Publicly available datasets were analyzed in this study. This data can be found here. This study uses publicly available, de-identified secondary data from the European Values Study (EVS) 2017 Trend File, accessible at https://search.gesis.org/research_data/ZA7503?doi=10.4232/1.14021.

## Appendix A: complete feature selection rankings

**Table A1:** Complete feature selection rankings for happiness (top 20).

Rank	EVS code	Description	Sel. Freq.	Stability	Consensus
1	A009_R	Health status	1.00	1.00	Yes
2	A173	Freedom/control	0.99	0.95	Yes
3	A002_R	Friends importance	0.80	1.00	Yes
4	X007	Marital status	0.80	1.00	Yes
5	E069_06_R	Confidence in police	0.69	0.86	Yes
6	A003_R	Leisure importance	0.67	0.84	Yes
7	G006_R	National pride	0.55	0.85	No
8	A165	Trust in people	0.54	0.83	No
9	X047_EVS	Income level	0.43	0.93	No
10	E036	Private vs state ownership	0.40	1.00	No
11	X011	Number of children	0.40	1.00	No
12	X003	Age	0.40	1.00	No
13	X023	Education	0.40	1.00	No
14	F025	Religious denomination	0.40	1.00	No
15	E035	Income equality	0.40	0.98	No
16	E039	Competition good/harmful	0.40	0.98	No
17	F063	God importance	0.40	0.98	No
18	E069_09_R	Confidence in social security	0.40	0.97	No
19	E037	Govt. responsibility	0.39	0.96	No
20	F118	Just.: Homosexuality	0.39	0.95	No

Sel. Freq., mean selection frequency across 100 outer iterations. Stability, selection stability index. Consensus, ≥60% of ensemble methods.

**Table A2:** Complete feature selection rankings for life satisfaction (top 20).

Rank	EVS code	Description	Sel. Freq.	Stability	Consensus
1	X007	Marital status	1.00	1.00	Yes
2	A173	Freedom/control	1.00	1.00	Yes
3	A009_R	Health status	1.00	1.00	Yes
4	G006_R	National pride	1.00	1.00	Yes
5	E069_06_R	Confidence in police	1.00	0.98	Yes
6	A080	Belong to none	1.00	0.98	Yes
7	E069_09_R	Confidence in social security	0.99	0.97	Yes
8	A165	Trust in people	0.99	0.96	Yes
9	X003	Age	0.90	0.88	Yes
10	F118	Just.: Homosexuality	0.87	0.88	Yes
11	A003_R	Leisure importance	0.85	0.89	Yes
12	F115	Just.: Avoiding fare	0.83	0.86	Yes
13	A002_R	Friends importance	0.82	0.82	Yes
14	X047_EVS	Income level	0.78	0.89	Yes
15	A001_R	Family importance	0.63	0.81	Yes
16	F063	God importance	0.51	0.87	No
17	E069_13_R	Confidence in companies	0.44	0.92	No
18	E037	Govt. responsibility	0.44	0.92	No
19	E069_07_R	Confidence in parliament	0.43	0.92	No
20	E035	Income equality	0.42	0.94	No

Sel. Freq., mean selection frequency across 100 outer iterations. Stability, selection Stability Index. Consensus, ≥60% of ensemble methods.
